# Molecular and Cellular Analysis of the Repair of Zebrafish Optic Tectum Meninges Following Laser Injury

**DOI:** 10.3390/cells11132016

**Published:** 2022-06-24

**Authors:** Payel Banerjee, Paul Joly, Luc Jouneau, Yan Jaszczyszyn, Mickaël Bourge, Pierre Affaticati, Jean-Pierre Levraud, Pierre Boudinot, Jean-Stéphane Joly

**Affiliations:** 1 TEFOR Paris-Saclay, UAR 2010, CNRS, INRAE, Université Paris-Saclay, Centre CEA, Bât. 151, 91400 Saclay, France; pierre.affaticati@cnrs.fr; 2 Ecole Normale Supérieure Paris-Saclay, 4 Avenue des Sciences, 91190 Saclay, France; pauljoly2000@gmail.com; 3 VIM, Université Paris-Saclay, INRAE, UVSQ, Domaine de Vilvert, 78350 Jouy-en-Josas, France; luc.jouneau@inrae.fr (L.J.); pierre.boudinot@inrae.fr (P.B.); 4 I2BC, CNRS, Université Paris-Saclay, 1 Avenue de la Terrasse, 91190 Gif sur Yvette, France; yan.jaszczyszyn@i2bc.paris-saclay.fr (Y.J.); mickael.bourge@i2bc.paris-saclay.fr (M.B.); 5 NII Group, Institut des Neurosciences Paris-Saclay, Université Paris-Saclay, CNRS, Institut Pasteur, Campus CEA, Bât. 151, 91400 Saclay, France; jean-pierre.levraud@pasteur.fr

**Keywords:** fish, midbrain, meninx, meningioma, arachnoid space, two-photon laser injury, ischemic accident, brain mild traumatic injury, radar plots, water channels, solute carriers

## Abstract

We studied cell recruitment following optic tectum (OT) injury in zebrafish (*Danio rerio*), which has a remarkable ability to regenerate many of its organs, including the brain. The OT is the largest dorsal layered structure in the zebrafish brain. In juveniles, it is an ideal structure for imaging and dissection. We investigated the recruited cells within the juvenile OT during regeneration in a Pdgfrβ-Gal4:UAS-EGFP line in which pericytes, vascular, circulating, and meningeal cells are labeled, together with neurons and progenitors. We first performed high-resolution confocal microscopy and single-cell RNA-sequencing (scRNAseq) on EGFP-positive cells. We then tested three types of injury with very different outcomes (needle (mean depth in the OT of 200 µm); deep-laser (depth: 100 to 200 µm depth); surface-laser (depth: 0 to 100 µm)). Laser had the additional advantage of better mimicking of ischemic cerebral accidents. No massive recruitment of EGFP-positive cells was observed following laser injury deep in the OT. This type of injury does not perturb the meninx/brain–blood barrier (BBB). We also performed laser injuries at the surface of the OT, which in contrast create a breach in the meninges. Surprisingly, one day after such injury, we observed the migration to the injury site of various EGFP-positive cell types at the surface of the OT. The migrating cells included midline roof cells, which activated the PI3K-AKT pathway; fibroblast-like cells expressing numerous collagen genes and most prominently in 3D imaging; and a large number of arachnoid cells that probably migrate to the injury site through the activation of cilia motility genes, most likely being direct targets of the FOXJ1a gene. This study, combining high-content imaging and scRNAseq in physiological and pathological conditions, sheds light on meninges repair mechanisms in zebrafish that probably also operate in mammalian meninges.

## 1. Introduction

The meninges encase the central nervous system (CNS) from its earliest stage of development and persist as a protective covering in the adult CNS [1]. The meninges may be directly or indirectly severed in diverse accidents, diseases, and surgical procedures. Their repair is important to prevent complications such as cerebrospinal fluid (CSF) leak. Tumors, such as meningiomas in particular, may also form in the meninges [1,2]. Better knowledge of the molecular mechanisms at work in the meninges is required to improve the management of all meningeal conditions.

The meninges consist of the dura mater, arachnoid (ARA), and pial layers [3]. They also contain fibroblasts, resident immune cells, and an extensive network of blood and lymph vessels. Large cerebral arteries course along the pial surface of the brain, giving rise to arterioles penetrating into the brain.

The meninges are involved in the development of the cranium and underlying brain and subsequently in many physiological processes contributing to brain homeostasis. Bone morphogenic proteins (BMPs) produced by the meninges help control cortical layer formation [4], whereas the retinoic acid produced by meningeal fibroblasts directs cortical neurogenesis and cerebrovascular development [5,6].

In mammals, the developing meninges are composed of the pachymeninx, which gives rise to the dura mater, and the leptomeninx, which gives rise to both the ARA and pial layers (Supplementary Figure S6) [7]. It is currently difficult to compare the complexity of the meninges in prenatal mouse embryos with that in juvenile zebrafish, because few reports on fish meninges have been published. Under the electron microscope [8], fish meninges can also be seen to consist of three layers: an outer layer possibly corresponding to the dura (‘pachymeninx’), an intermediate layer with putative ARA, and an inner layer (‘leptomeninx’) with vessels and a basement membrane.

In this manuscript, we first focus on the cellular composition of the meninges of zebrafish (*Danio rerio*). The zebrafish model, with its considerable regenerative capacity, has made a major contribution to our understanding of the cellular and molecular mechanisms underlying organ regeneration. This model species has been particularly useful for studies of organs with limited regeneration potential in mammals, such as the heart [9,10,11,12] and nervous system [13,14]. We studied fish 21 days post-fertilization (dpf), because at this stage it is easy to dissect the brain, to analyze reasonably mature immune and nervous systems, and to perform rapid and easy three-dimensional (3D) imaging of the brain, which is not covered dorsally by a thick cranium as it is in adults. We followed the outcome of injury to the optic tectum (OT), as this large dorsal region of the brain is the easiest to image in live or fixed samples. We focused on cells expressing platelet-derived growth factor receptor beta (PDGFRβ), which is known to be expressed on pericytes playing a key role in blood vessel integrity, and in the lymphatic vasculature of the meninges [15]. We used an existing BAC-derived transgenic zebrafish line faithfully driving Gal4 expression in PDGFRβ-expressing cells [16]. We first used 3D imaging and single-cell RNA sequencing (scRNAseq) to characterize the cell types expressing EGFP, and to describe the meningeal cell types over and around the OT. In the spinal cord, PDGFRβ-positive fibroblast-like cells deposit regeneration-promoting Col XII in a Wnt signaling-dependent manner at lesion sites [17].

We first settled three methods of injury of the OT. Needle injury led to very variable outcomes such as fast closure or abnormal regeneration. Deep laser injury did not lead to massive recruitment of any PDGFRβ/EGFP-positive cells, including pericytes. We then investigated OT regeneration following laser surface injury, which creates a meninx/brain blood barrier (BBB) breach. To our surprise, following such injury, we consistently observed EGFP-positive cells converging on the wound at the surface of the midbrain in the so-called arachnoid space [18,19]. One day post-injury (dpi), our scRNAseq analysis revealed the recruitment of diverse cell types: ligament cells, chondrocytes, pial, fibroblastic, ARA, dural, and MID cells. We then focused on the ARA cells, which overexpress the Aqp1a.1 gene during regeneration. During migration to the wound, ARA cells also overexpressed genes from several gene families involved in motile cilium mediating cell motility.

## 2. Materials and Methods

### 2.1. Zebrafish Husbandry and Transgenic Lines

Eggs obtained by natural spawning were bleached and incubated at 28 °C in embryonic medium (EM) [20]. Zebrafish embryos were reared according to standard procedures, as previously described [20]. Wild-type (WT) and *casper* zebrafish were obtained from ZIRC (Eugene, OR, USA) or EZRC (Karlsruhe, Germany). The environmental parameters were as follows: photoperiod = 14 h light/10 h dark, temperature = 26.5 ± 1 °C, pH = 7.8 ± 0.1; conductivity = 240 ± 30 μS/cm, [NH_4_^+^] = 0 mg/L, [NO_2_^–^] = 0 mg/L, [NO_3_^–^] < 50 mg/L. Fish were fed with rotifers (*Brachionus plicatilis*, ~500/fish/day) for two weeks, and then with brine shrimps (*Artemia nauplii*, ~250/fish/day) and dry food (Skretting, Gemma Micro, twice daily). All protocols were approved by the local ethics committee for animal experimentation (CEEA 59) and the French Ministry of Research and Education. All procedures were performed in accordance with European Union Directive 2011/63/EU, with the approval of the local ethics committee (no. 59 CEEA). The lines used in this study were TgBAC(Pdgfrβ:gal4FF)^ncv24^, Tg(5×UAS;EGFP)^nkuasgfp1a^, Tg (kdrl:DsRed) [16]. Transparent juveniles with strong EGFP expression were obtained by performing crosses to generate homozygous transgene insertions in a *casper* background [21]. Some Upstream Activating Sequences (UAS)-driven transgenes are subject to silencing problems. The breeding stock for this study was therefore screened to select the animals with the highest levels of expression in their progeny.

### 2.2. Three Types of OT Injury

We prepared a stock solution of 10× ethyl 3-aminobenzoate methanesulfonate (MS222) for anesthesia by dissolving 400 mg of tricaine powder and 800 mg sodium bicarbonate in 200 mL EM [20]. Before mounting, 21 dpf zebrafish were transferred from their tanks to a Petri dish containing 0.5× MS222 solution. Anesthesia treatment was stopped after 45–60 s, when the fish stopped swimming, and 7.8 mm/21 dpf juveniles were mounted in aligned wells in agarose plates. These wells were obtained by placing 3D-printed plastic molds with 12 wells in negative, mimicking juvenile body shapes, on top of 1% agarose. These wells prevented most of the movements of the juveniles. Fish movement was further prevented by applying a drop of 1.5% low-melting point agarose (LMA) at the tail end. The LMA took about 10 s to harden at normal room temperature (20 °C to 25 °C). The plate was then filled with EM containing 0.5× MS222 during the brain injury process. Plastic Pasteur pipettes and paint brushes were used to transfer the fish to 3D printed wells and to orient them dorsally. The fish were injured, as described below, and transferred to fresh fish embryonic medium. Air bubbles were applied with a pump to help the juveniles recover. After recovery, the fish displayed normal swimming and feeding behavior.

#### 2.2.1. Deep Multiphoton Laser Injury

The center of the OT was burnt with a laser in a parallelepiped volume of 88 × 88 × 100 µm (*x*,*y*,*z*) at a depth of 100 µm into the brain tissue. A Leica TCS SP8 MP multiphoton microscope equipped with a 25× objective was used for laser injury. The biphoton laser was tuned to a wavelength of 750 µM, and a power of 2.5 mW with an intensity of 50% and an offset of 45%. These parameters were used for the injury of the selected area with a 2 µm z-step, in bidirectional scanning mode, with a pixel size of 2 µm. The injured juveniles were revived according to the procedure described in Section 2.3.

#### 2.2.2. Needle Injury

Under a dissecting microscope, a minute needle (200 µm diameter) was inserted vertically through the skull into the medial region of the OT at a depth of 300 µm. 

#### 2.2.3. Surface Multiphoton Laser Injury

Sample handling before and after injury and laser parameters were identical to those described in Section 2.2 and Section 2.2.1. We focused the laser on the center of the surface of the OT volume. The location of the injury on the *z* axis was determined by selecting the optical section over the skin surface to define the upper part of the injury and moving 100 µm deeper into the tissue.

### 2.3. Live Imaging of Zebrafish Larvae

For live confocal imaging, juvenile zebrafish were anesthetized in EM medium containing 0.5× MS-222 and mounted in the appropriate orientation in 1.5% low-melting point agarose (Ultrapure Low Melting Point, Invitrogen, Waltham, MA, USA). A mounting plate was prepared with dentistry silicone (Picodent, Wipperfürth, Germany) and a negative 3D-printed plastic mold. During imaging, juveniles were covered with EM containing 0.5× MS-222. Imaging was performed with a Leica SP8 confocal microscope equipped with a water immersion objective (25×). Pixel size was 1.16 µm × 1.16 µm (along the *x* and *y* axes) and 2 µm along the *z* axis. Stacks of about 300 µm were acquired. The duration of each experiment at each time point was kept as short as possible—typically 25 to 30 min, including about 10 min of imaging—to ensure that the zebrafish juveniles remained alive and efficiently recovered. The area of each imaged sample could be iteratively identified at successive time points according to the Kdrl-DsRed pattern. After each acquisition, the juveniles were released from the low-melting point agarose, revived in fresh aerated tank water, and then returned to the facility tanks until the next acquisition.

### 2.4. Immunohistochemistry

We adapted classical antibody protocols for the staining of meningeal cells from 21 dpf zebrafish without cell destruction and minimizing the background. Immunohistochemistry (IHC) was performed with a higher than usual percentage of saponin to allow penetration through the skin. High dilutions of antibodies and short incubation times were used to prevent excessive background staining.

The 21 dpf juveniles were killed by immersion in 10× MS-222 in phosphate buffer saline (PBS). Samples were then fixed by incubation in freshly prepared 4% PFA-PBS for 30 min at room temperature (RT), rinsed quickly with 0.1% Tween 20 in PBS (PBST), and washed six times, for five minutes each, in PBST at RT with gently shaking. The samples were incubated with blocking reagent (10% NGS, 10% DMSO, 1% Triton X-100, 0.1% Tween 20, 0.5% saponin in PBS) for two hours at RT before immunostaining. They were then incubated overnight at RT with primary antibodies (anti-Aqp1a.1 antibody 1:250 [22] and anti-GT335 1:1000 [23]) in fast staining solution (2% NGS, 20% DMSO, 0.1% Triton X-100, 0.1% Tween 20, 10 µg/mL heparin, 0.5% saponin in PBS), with gentle shaking. Samples were then washed six times in PBST, for 10 min each, with gentle shaking, and were incubated with the appropriate fluorescent secondary antibodies in fast staining solution for 4 h at RT. Throughout this process, the samples were kept in complete darkness. Finally, the samples were thoroughly washed six times, for 10 min each, in PBST, with shaking. For image acquisition, samples were placed in a high-refractive index clearing medium (adjusted to a refractive index of 1.457). We added 225 g sucrose, 100 g nicotinamide, 50 g triethanolamine, 500 µL Triton X-100, and 500 mL double-distilled water 500 mL for each 100 mL of medium. Successive incubations were performed in 25%, 50%, 75%, and 100% clearing medium, for 30 min each, with gentle shaking. Samples were imaged with a Leica laser scanning confocal microscope equipped with 25× and 40× objective objectives for high resolution. The images were analyzed with Amira (2019) and ImageJ software.

### 2.5. Cell Dissociation

We dissected ten OTs and collected them in an Eppendorf tube placed in ice and filled with cold PBS supplemented with 5% Bovine Serum Albumine (BSA). The dissection step had to be performed sufficiently quickly to prevent cell degradation. The dissected OTs were transferred to 400 µL of dissociation buffer (Miltenyi Biotec, Bergish Gladbash, Germany. Cells were dissociated by gentle trituration with a glass pipette. The pipette was iteratively filled with the dissociation buffer and pieces of dissected OTs, which were then released into the Eppendorf tube. After trituration, the sample was centrifuged at 4 °C at 300× *g* for ten minutes. The supernatant was discarded, and the pellet was resuspended in 1 mL cold PBS. The suspension was passed through a sterile filter with 50 µm pores to remove clumps of cells before introduction into the cell sorter.

### 2.6. Sample Preparation for Cell Sorting

FACS was performed with a slightly modified version of a published protocol for zebrafish juveniles (Supplementary Figure S4) [24]. Isolated cells were sorted by flow cytometry with a MoFlo Astrios_EQ© cytometer (Beckman Coulter, Roissy, France), in PuraFlow 1× sheath fluid (Beckman Coulter) at 25 psi (pounds per square inch), with a 100-micron nozzle. We performed sorting with a drop drive frequency of ~43 kHz, plate voltages of 4000–4500 V, and an amplitude of 30–50 V. Sorting was performed in purity mode. The 488-SSC (side scatter) parameter was set as the threshold. Cell gates were first set on the FSC-area (forward scatter) versus SSC-area plot. Doublets were discarded by gating on the SSC-area versus SSC-height plot. A DAPI-positive gate was also used to exclude dead cells. Finally, EGFP-positive cells were selected for sorting and compared with EGFP-negative samples. Gating accuracy was determined by post-sorting microscopy. The flow cytometer-sorted cells (10 to 20 thousand cells) were collected in 1.5 mL tubes for sequencing.

### 2.7. Single-Cell RNA Sequencing (scRNA-seq)

After FACS, the cells were collected in 1×PBS-like medium (pH 7.4) at a density of 300 cells/µL in 50 µL. Because only 30 µL could be loaded in the 10× Genomics chip, 9000 cells (with a target recovery of 6000 cells) were used with a ‘Chromium Next GEM Single Cell 5′ V2’ kit. We generated cDNAs with a Chromium Controller, according to the manufacturer’s recommendations. Libraries for Illumina sequencing were prepared according to the manufacturer’s recommendations, and approximately 6000 cells were sequenced on an Illumina NextSeq 550, with paired end 26–134 bp sequencing runs. Raw reads were aligned to the Zebra_Ensembl_102 version of the zebraFish genome GRCZ11 from Ensembl [25] using the Cell Ranger 3.1.0 pipeline. The EGFP sequence was added to the annotated genome to perform alignments. Experiment 1 generated 2956 cells originating from control juveniles and 3025 cells from injured fish. Similarly, experiment 2 generated 2085 and 3019 cells.

### 2.8. Quality Control, Batch Correction, and Doublet Removal

First, a single dataset from two control samples arising from two independent experiments was created using the merge() function for cell heterogeneity analysis. Second, another dataset was generated including cells from control and injured fish from the two same independent experiments. Because the same protocol was followed in the two experiments, we considered them as technical replicates. For each analysis, cells were selected if they had (1) between 100 and 7500 features expressed, (2) between 1500 reads and 50,000 reads, (3) less than 2.9% of mitochondrial genes, and (4) more than three EGFP reads. These parameters identified 575 and 448 cells for control fish and 603 and 597 cells for the injured fish. Following quality control, all downstream analyses were implemented using the R package Seurat V4 from the Satija lab [26]. Data were normalized using the NormalizeData() function using the LogNormalize method with a scale factor of 10,000. The 2000 top variable genes were selected using the FindVariableFeatures() function. A standard scaling with no regression was done using the function ScaleData(). PCA were calculated on the Top 2000 variable genes using the RunPCA() function. A significant amount of overlap between datasets was observed on the Uniform Manifold Approximation and Projection (UMAP) plot of the merged analysis for control experiments and for control-injured experiments (Supplementary Figure S1B). This suggested that dataset merging did not lead to a batch effect.

As we were focusing on sticky meningeal cells, we eliminated any suspected doublet cells in the control-injured experiment. Doublets were identified using the DoubletFinder [27] method guideline with a predicted proportion of 30% of total cells and using ten principal components (PCs). Similar numbers of cells were removed in all experiments, leading to a merged dataset of 1556 cells.

Finally, two datasets were created, a first one for tectum cell heterogeneity analysis with non-injured EGFP-positive brain cells, and a second one for cell recruitment during injured response analysis with non-injured and injured EGFP-positive brain cells.

### 2.9. Cluster Identification and Annotation

Unsupervised clustering was performed with the Seurat FindClusters() function with a resolution of 0.6 for OT cell heterogeneity analysis and a resolution of 1.5 for cell recruitment analysis. UMAP plots were generated with 30 PCA for the first analysis and 20 for the second one according to the Jackstraw analysis. For the second experiment, subsets were generated with control or injured status. Differentially expressed genes (DEGs) of each cluster were identified using the FindAllMarkers() function.

### 2.10. Gene Ontology Analysis

The Gene Ontology analysis was performed with the Metascape Resource [28]. For the analysis of enriched gene families in pooled cells from the two experiments with control fish, GO terms were identified from the first 100 differentially expressed genes (DEGs) of each cluster with the highest fold-changes of expression levels. For the control-injured analysis, DEGs were sorted into three categories: first, ‘controls’ with FC > 0.25; second, ‘common’ control- and injured-enriched’, both with FC > 0.25 and in addition with a greater enrichment in cells from injured fish than those arising from controls (DeltaFC > 0.25); third, ‘injured-specific’ DEGs were selected if they were present specifically in the injured fish list (FC > 0.25) following a Venn diagram also including the list of genes from control fish cells (FC > 0.25).

## 3. Results and Discussion

### 3.1. The PDGFRβ-GAL4:UAS-EGFP Line Has a High Diversity of EGFP-Positive Cells

#### 3.1.1. Main Populations of EGFP-Positive Cells

**Figure 1:**
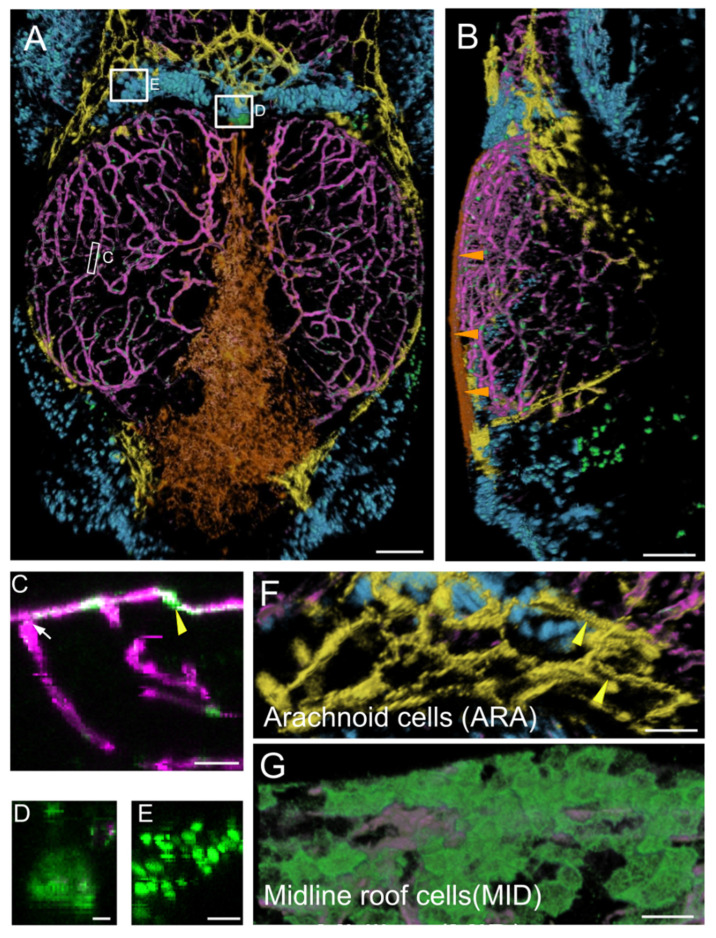
Main expression domains of the PDGFRβ-gal4:UAS-EGFP line as seen by high-content confocal imaging of 21 dpf juveniles. Three main meningeal domains were segmented and color-coded in orange, yellow and blue. (**A**,**B**). 3D reconstructions using the AMIRA software. Midline roof cells (MID): orange; arachnoid cells (ARA): yellow; red blood cells (RBC): blue; vessels: magenta. (**A**). Dorsal view. White rectangles indicate locations of details in (**C**–**E**). (**B**). Lateral view. Orange arrowheads show the flat MID. (**C**). XZ thick optical section showing one pericyte (yellow arrowhead) and one vessel (white arrow). (**D**). Dorsal view of choroid plexus. (**E**). Red Blood Cells (RBC). (**F**). Dorsal view of ARA showing typical elongated and square shapes (as described for mammalian interstitial arachnoid cells) and granulations (yellow arrowheads). (**G**). Dorsal view of MID stained with Aqp1a.1 antibody showing their round and flat ameboid-like shape. Scale bars: (**A**,**B**): 100 μm. (**C**–**G**): 50 μm. (**D**–**E**): 10 μm.

We visualized *PDGFRβ*-expressing cells in the brain vascular system by generating triple homozygous transgenic TgBAC (Pdgfrβ:gal4FF)^ncv24^, Tg(5× UAS;EGFP)^nkuasgfp1a^, Tg (kdrl:DsRed) *casper* pigment-depleted juveniles. EGFP-positive cells were found in many domains (Figure 1A,B). We obtained high-content images by clearing the juveniles and mounting them in high-refractive index media. Acquisition was performed on confocal microscopes equipped with dipping lenses with long working distances (see Materials and Methods).

We identified six domains of EGFP-positive cells on the basis of cell distribution and shape. The first three prominent ones were not segmented for 3D viewing because of their widespread distribution in the OT or their location deep in the tissue.

As expected, the first category of cells identified was the pericytes, which were tightly affixed to the vascular endothelium and were strongly labeled (Figure 1C). More surprisingly, we detected a second domain of mild EGFP expression corresponding to endothelial cells (Figure 1C). Vessels were present throughout the midbrain, forming a large network at the surface of the brain connected to the peripheral meningeal vessels and to vessels penetrating the brain (Figure 1A–C). A third category of EGFP-positive cells was found in the choroid plexus (Figure 1D), at the border between the forebrain and midbrain, deeper within the brain than the meninges.

A prominent group of positive cells was located between the OT and telencephalon. These cells formed a very large population of small round cells (Figure 1E), color-coded in cyan in Figure 1A. Another similar population of small round cells was found in the large dorsolateral spaces between the OT and the cerebellum. Two clusters of EGFP-positive blood cells (red blood cells (RBC) and thrombocytes) were identified in our transcriptomic approach (see below). We therefore suggest that these cells may be RBC/thrombocytes located in venous sinuses known as cisterns. It has been suggested that these structures drain cerebrospinal fluid (CSF) via arachnoid granulations following the one-way circulation of the CSF through the subarachnoid space after its production in the choroid plexus [29,30,31].

By segmenting and visualizing cells with Amira, we generated a detailed description of two new cell populations located in the meninges of the dorsal midbrain:

- Midline roof cells (MID), color-coded in orange. These cells were found between the telencephalon and cerebellum (Figure 1A,G) at the most dorsal part of the brain midline. They were very flat and closely packed together, particularly over the cerebellum, as shown in an *xz* thick optical section (Figure 1B).
- Arachnoid cells (ARA), color-coded in yellow. This population of cells with strong, positive EGFP staining was located in the so-called arachnoid space [32,33] at the periphery of the OT, at the surface of the cisterns [29,30,31], adjacent to the telencephalon and cerebellum (Figure 1A,G). In mammals [18,19], the subarachnoid space also extends between the telencephalon and midbrain, and between the midbrain and cerebellum. These EGFP-positive cells in the arachnoid space clearly did not constitute a homogeneous barrier at this stage. The cells had a typical rectangular elongated shape (Figure 1), as reported for the interstitial epithelial cells of the arachnoid in rat cell cultures [34]. Some of these EGFP-positive cells presented granulations resembling those of mammalian arachnoid cells (Figure 1G) [35].

#### 3.1.2. Diversity of EGFP-Positive Cells Captured by scRNA-seq

**Figure 2:**
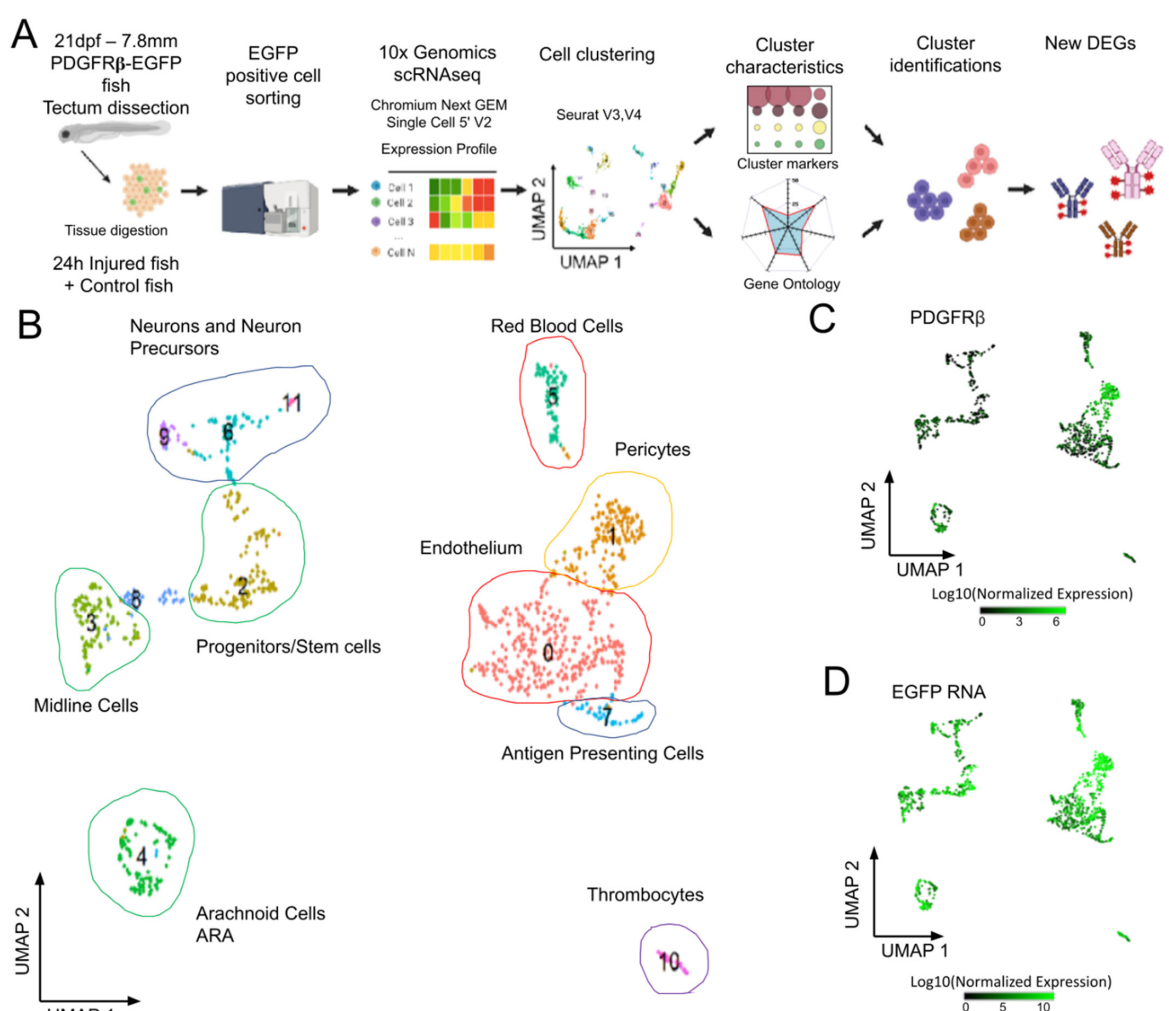
Analysis of scRNAseq data highlights cell heterogeneity of GFP positive cells from dissected tecta of PDGFRβ-gal4:UAS-EGFP juveniles. (**A**). Experimental workflow for the collection and isolation of OT cells. (**B**). Uniform manifold approximation and projection (UMAP) showing cell populations from control 21 dpf juveniles. The projection was performed with the 30 first principal component analysis of gene expression. Each point represents a single cell with a colour indicating its membership to a cluster. 0: KDRL- Endothelium; 1: NDUFA.4L2A-Pericyte; 2: RPS2-Progenitors, Stem cells; 3: ZIC4- Midline Cells; 4: AQP1A.1-Arachnoid Cells; 5: HBAA1-Red Blood Cells; 6: ELAVL4- Neurogenic Precursors; 7: CD74A-Antigen Presenting Cells; 8: Uncharacterized; 9: NEUROD1- Neurons and Precursors; 10: THBS1B- Thrombocytes; 11: HPCA- Neurons. (**C**). Featureplot highlighting PDGFRβ transcript levels in cells. (**D**). Featureplot of GFP transcript levels. PDGFRβ expression is correlated to GFP showing that most GFP+ cells express PDGFRβ.

Microscopy clearly showed that the zebrafish transgenic PDGFRβ reporter line typically displayed pericyte labeling, but that EGFP was also expressed by several other cell types, notably in the meninges. We investigated the diversity of EGFP-positive cells by performing scRNAseq analysis on FACS-sorted EGFP-positive cells in physiological/control conditions (Figure 2A).

OT from 21 dpf PDGFRβ-Gal4:UAS-EGFP juveniles were dissected and processed. Data from two independent experiments were merged to form a single dataset for 3483 cells to make it possible to assess the reproducibility of the results. We retained cells with more than three EGFP hits (1129 cells) for further cell cluster analysis. Principal component analysis followed by uniform manifold approximation (*UMAP*) analysis identified twelve cell clusters (Figure 2B). Clusters were identified on the basis of previously reported DEGs, and/or through GO analyses [28]. As expected from imaging data, the cluster with the highest level of PDGFRβ and EGFP expression (cluster 1, Figure 2C,D) corresponded to pericytes (C1, PDGFRβ, ANGPTL3, NOTCH 3, FOXC1B, and KCNJ8 CSPG4 RGS5 CD248 ABCC9 [36]). More surprisingly, we detected endothelial cells with medium levels of EGFP expression (cluster 0, Figure 2D) and DEGs typical of this cell type (e.g., C0, KDR, KDRL, FLI1A).

Three small cell clusters were found to correspond to hematopoietic cells, circulating blood cells, for two of these clusters. Cluster 5 corresponded to red blood cells (RBCs) (Figure 2, Supplementary Figure S1A) expressing hemoglobins and carbonic anhydrase (HBAA1, CREG1, NT5C2L21, etc.) (Supplementary Table S1). These RBCs were probably mature RBCs, rather than precursors, as GATA1 was downregulated in these cells. Cluster 7 contained antigen-presenting cells (APCs) (Figure 2 and Figure S1A) expressing CD74a/b, B2M, MHC II, and IRF8 (Supplementary Table S1). Their high levels of APOEB expression suggested that they were microglia, but the low levels of MPEG1.1, SPI1a, AKA, PU.1, CSF1r, and SLC7a expression were not consistent with the published transcriptome of these cells [37]. Some of the top-ranking DEGs, such as the CFCL34B.1 and IFI44D genes, were typical ISG expressed in interferon-stimulated larvae [38], suggesting that the type I IFN pathway was activated. Many of the GO terms displaying enrichment in these cells were common to those displaying enrichment in cluster 10 (Supplementary Figure S1A), which corresponded to thrombocytes expressing ITGA2b and TBS1b (Supplementary Table S1). Another cluster included potential stem cells (C2). These cells had high levels of expression for genes encoding elongation factors, ribosomal proteins, and proteins involved in ribosome biogenesis (NOP53, NOP56, etc.) (Supplementary Table S1). A cluster of proliferating cells of unknown type was also present (C8). Finally, our GO analysis grouped three clusters of neuronal cells together on the basis of function (C6, C9, C11) (Supplementary Figure S1A).

Our analysis at homeostasis provides a detailed description of the cell types found in the PDGFRβ-GAL4:UAS-EGFP transgenic line. It shows that it will be interesting in the future to use this line to study immune and proliferating cells in the OT. The clusters identified in this study are different from those reported in a previous study [39]. This discrepancy may reflect the many differences between the two studies: stage (juvenile versus larva), region (dissected OT versus whole body), PDGFRβ-GAL4:UAS-EGFP versus PDGFRβ-GFP. Moreover, the authors of this previous study reported the presence of EGFP-positive cells in EGFP-negative domains, whereas we observed a strong correlation between PDGFRβ and EGFP expression (Figure 2C,D).

### 3.2. Studying Regeneration in Juveniles after OT Injury

#### 3.2.1. Three Injury Procedures Result in Very Different Outcomes

**Figure 3:**
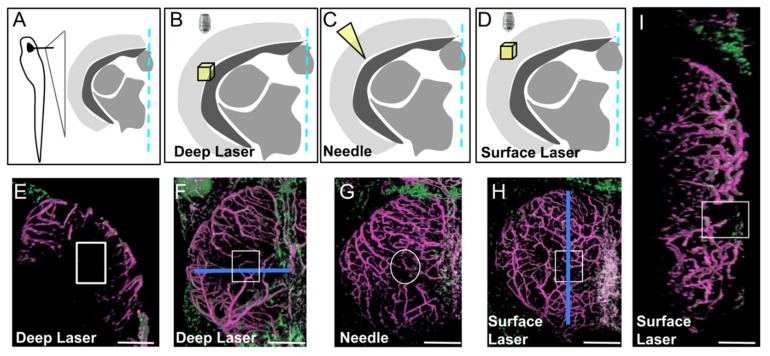
Three types of injury of the optic tectum (OT) at 21 dpf. (**A**). Schematic cross section at the level of the center of the OT with approximate location of details in (**B**–**D**). (**B**,**E**,**F**). Deep biphoton laser injury. (**C**,**G**). Needle injury. (**D**,**H**,**I**). Surface biphoton laser injury. Blue lines in (**F**,**H**) indicate locations of sections shown in (**E**) and (**I**). (**E**,**I**). Cross and parasagittal sections showing locations of wounds (white rectangles). (**F**–**H**). Dorsal views with location of injuries (white squares and circle). Anterior at the top. Midline on the right. Scale bars: (**E**–**I**): 100 μm.

We created three types of injuries (see Materials and Methods) resulting in the defects shown in Figure 3, Figures S2 and S3.

**Figure 4:**
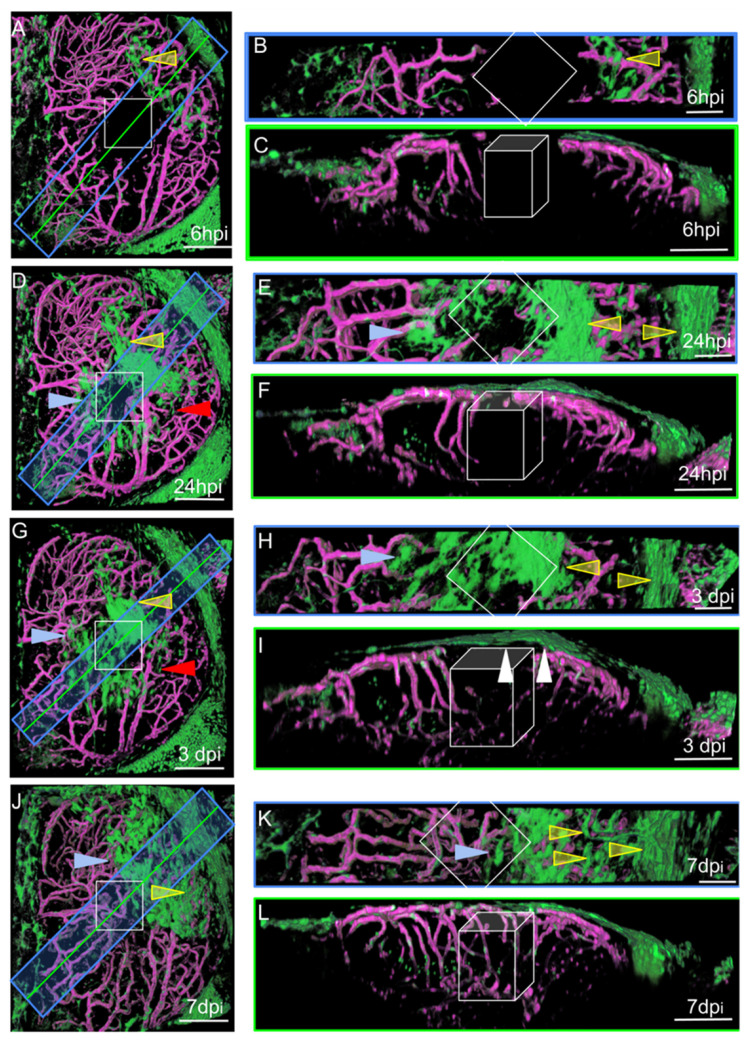
Surface laser injury of the optic tectum (OT) at 21 dpf. (**A**,**D**,**G**,**J**). Dorsal views. Blue rectangles: locations of horizontal sections in the right panels. Yellow lines: locations of XZ sections in the right panels. Anterior at the top. Midline on the left. (**B**,**E**,**H**,**K**) Horizontal thick sections. (**C**,**F**,**I**,**L**) XZ sections. (**I**). At 3 dpi, multiple layers of arachnoid cells are visible over the wound (white arrowheads). Putative injured zones: white squares and boxes. ARA-like cells: yellow arrowheads. MID-like cells: blue arrowheads. Fibroblast-like cells: red arrows. Scale bars: (**A**–**L**): 100 μm. (**B**,**E**,**H**,**K**): 50 μm.

Needle injury resulted in highly variable wound repair (Supplementary Figure S2), probably depending on the depth of the needle puncture. For injuries limited to the OT layers with no ventricle disturbance, wound repair was extremely fast, sometimes occurring within one dpi (Supplementary Figure S2D). For deeper punctures, disorganization was highly variable, and regeneration was abnormal. We therefore chose to create injuries with a biphoton laser, this technique having the additional advantage of leading to scar formation, thereby better mimicking ischemic cerebral accidents. Despite extensive investigations of cell recruitment after deep laser injury, only a few EGFP-positive cells were observed at the wound. As in homeostatic conditions, strongly EGFP-positive pericytes were found solely associated with regenerating vessels (Supplementary Figure S3). Finally, we chose to perform laser injury at the surface of the OT to create a BBB/meninx breach. Surprisingly, injuries of this type resulted in the massive recruitment of EGFP-positive cells strongly expressing EGFP throughout the wound at one dpi (Figure 4).

#### 3.2.2. ARA-like Cells Respond to Injury by Forming Multiple Layers over the Wound

We performed live imaging of the OTs of individual wounded zebrafish at various times post-injury. At each time point, a Z-stack was acquired, and the animal was then allowed to recover for a few hours before repetition of the imaging procedure. This approach generated pseudo-time lapses with intervals of several hours, or even days, between images. The characteristic distributions and shapes of EGFP-positive cells made it possible to follow their movements, at the population level, with confidence l. At 6 hpi, MID and ARA cells were found to be dispersed over the OT around the wound (Figure 4A).

The cells originating from the midline were ameboid in shape, whereas those from the periphery were large, elongated, and rectangular. Small fibroblast-like cells were also observed and were not associated with vessels. No such cells were detected in control fish, suggesting that they probably proliferated after injury, as indicated by scRNAseq experiments (see below). One day after injury, most of the cells collected together around the wound and began to form densely packed multilayers to favor wound closure (Figure 4D). At 3 dpi, the cells continued to accumulate over the wound (Figure 4G). Vessel regeneration occurred and appeared to be complete by 7 dpi (Figure 4J). At this time point, the EGFP-positive cells formed a continuous single layer over the wound, and it was not really possible to distinguish between the cells originating from the midline and those originating from the peripheral meninges (Figure 4L). Our observations are consistent with a scenario in which meningeal EGFP-positive cells detach from the periphery of the OT to repair and repopulate the lesion site after injury.

#### 3.2.3. Three Clusters Potentially Recruited for Meninx Repair

Following on from our microscopy observations, we focused our scRNAseq study on potential meningeal cell types, beginning with a comparison with mammals.

**Figure 5:**
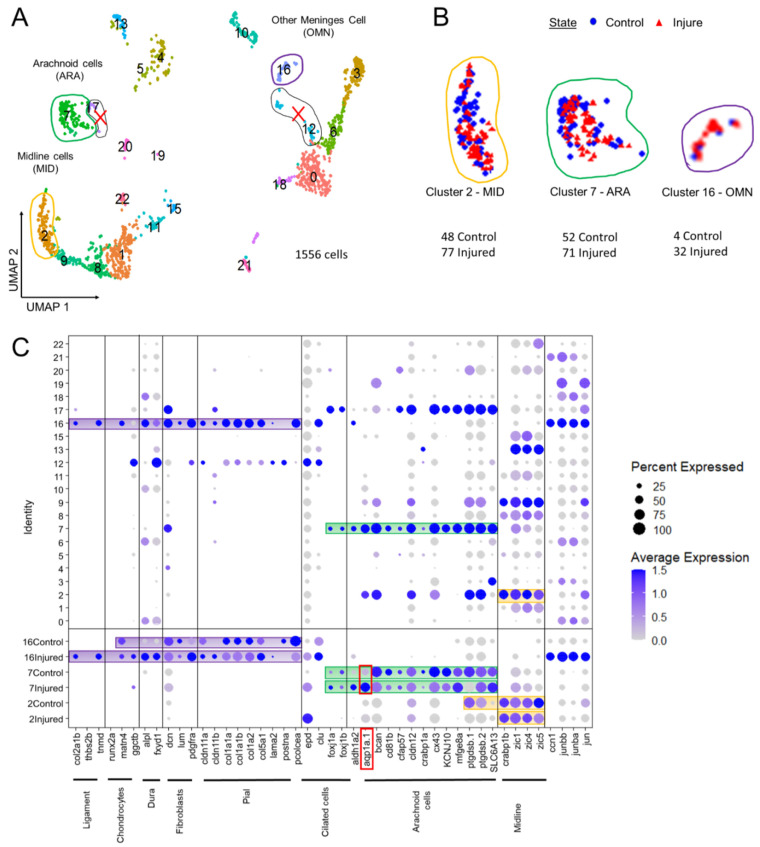
Analysis of scRNAseq data highlight cell heterogeneity of GFP cells from dissected tectum of PDGFRβ-gal4;UAS: EGFP in 21 dpf injured fish and 21 dpf control fish. (**A**). Uniform manifold approximation and projection (UMAP) showing cell populations from 21 dpf control and injured fish. The projection was computed from the twenty first principal component analyses of gene expressions. Each point represents a single cell with a colour indicating its membership to a cluster. (**B**). UMAP of injured and control cells colored according to their sample types (blue circle for control fish and red triangle for injured fish). Approximately equal numbers of cells of each origin was observed in most clusters. This avoided batch effect. (**C**). Cluster analysis of characteristic DEGs from different cell populations including arachnoid cells (ARA) found in cluster 7, midline-like cells (MID) found in cluster 2 and other meninx cells (OMN) found in cluster 16. Cluster 17 et 12 contained DEGs similar to clusters 2, 7, 16 but these clusters were discarded from our analysis due to their small size and heterogeneity.

As a means of identifying the cell types recruited after injury, we merged the data obtained from two previous experiments on 21-day-old control fish (10 dissected OT, 781 EGFP-positive cells) with data from two experiments performed on fish one day after surface laser injury (10 pairs of dissected OTs with a single OT injured, 774 EGFP-positive cells). The UMAP revealed 22 clusters, each composed of about half control cells and half cells from samples obtained 24 h post injury (hpi) (Figure 5B).

We used two recent transcriptomic studies of murine E14 meninges [40] and of cranial sutures [41] as guides for selecting DEGs, together with a review [42] listing the diverse types of meningeal cells (Figure 5C). We were therefore able to identify zebrafish clusters containing DEGs that were orthologs or members of co-orthologous sets of mouse meningeal genes. We identified five main clusters containing meningeal DEGs (Figure 5A). We excluded two very small clusters (12 and 17) from the analysis even though they contained ARA and pia DEGs. We selected three clusters for further analysis: cluster 2 (candidate midline roof cells, MID); cluster 7 (candidate arachnoid cells, ARA); and cluster 16 (other potentially meningeal cell types (OMN)) (Figure 5C).

#### 3.2.4. Clusters Containing Potential Midline (C2, MID) and Arachnoid (C7, ARA) Meningeal Cells Have Similar Patterns of DEG Enrichment, but Also Specific Hallmarks

A very large number of genes—550 of the 1416 C7 genes and the 1111 C2 genes—were found to be enriched (FC > 0.25) in both these clusters (Supplementary Table S2).

**Figure 6:**
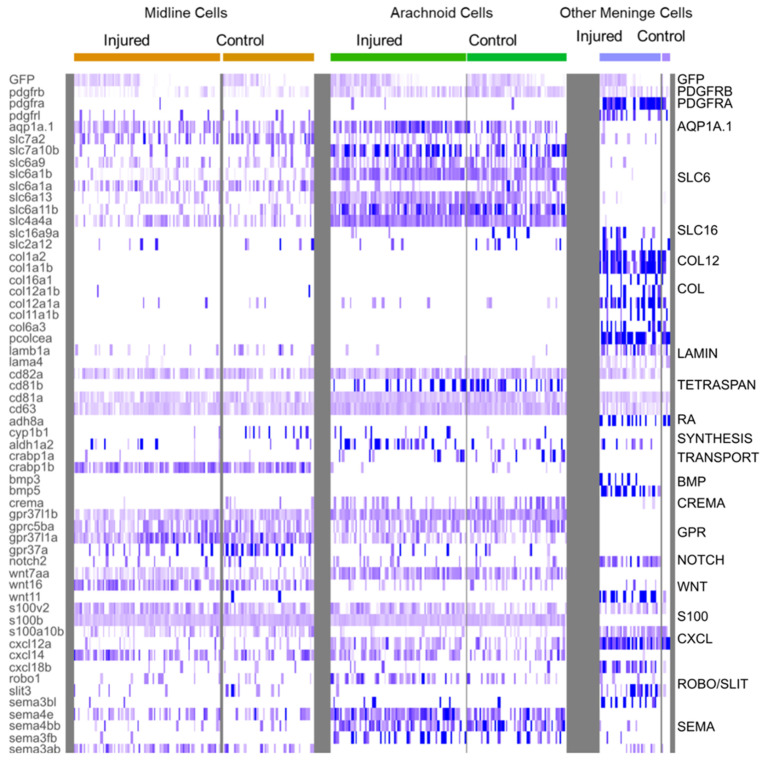
Heatmap of characteristic gene expression levels in MID, ARA and OMN cells from control and injured datasets. Each column represents a cell. Level of expression is encoded by color gradient. Strong expression is in dark blue and no expression is in white. Gene families are represented to the right of the panel in regards to the corresponding DEGs.

Various Wnt signaling molecules were expressed in cluster 2 (e.g., Wnt16) and cluster 7 (e.g., WNT7AA) (Figure 6). Wnt signaling may, thus, play a major role in repair, as in the spinal cord [17]. The sub-functionalization of the tissue-specific expression of CRABP1A and CRABP1B, two paralogs encoding retinoic acid transporters, has been reported in zebrafish [43]. We showed that cluster 7 displays an enrichment in CRABP1A, whereas cluster 2 cells strongly express CRABP1B [43] (Figure 6). Cluster 2 appeared to be composed of midline roof plate cells (MID). Indeed, the Zic1 and Zic4 genes displayed strong enrichment in cluster 2 (Figure 5C). These genes are known to specify the midline roof plate during zebrafish development [44,45]. Other genes strongly expressed at the dorsal midline at 3 dpf, as shown by in whole-mount in situ hybridization (WMISH) data from the Zfin database, were found at the top of the list of enriched genes (Supplementary Figure S5A–L). One notable exception to the massive enrichment in collagen gene expression in another candidate meningeal cell cluster (C16, see below) was the strong expression of col5a2a in MID (Figure 6).

Cluster 7 probably corresponds to arachnoid cells (ARA). Indeed, many DEGs typical of mouse ARA pathways, such as solute carrier (SLC) influx transporter proteins, were found in this cluster (Figure 6), together with fish orthologs of mouse genes expressed in the arachnoid (Figure 5C). In the meninges, the ARA layer expresses tight junction coding genes (Cx43, CLDN12, CDH2). Thus, in mammals, the ARA layer provides a selective barrier between the dura and the CSF-filled brain. ARA interstitial cell transcriptomes were highly enriched in water channels (Aqp1a.1), potassium channels (KCNJ10), solute transporter Slc6a13 (GABA transporter), and many other SLC transporters (SLC1A2B, SLC1A3B, SLC3A2A SLC4A4, SLC6A1B, SLC6A9, SLC6A10, SLC7A10B, SLC27A1B) (Figure 6). An enrichment in many transmembrane proteins, such as CD81b (proteins encoding transmembrane 4 superfamily/tetraspanin members) was observed (Figure 6). ARA were found to be enriched in DEGs involved in meningioma formation, such as the prostaglandin synthase PTGDS.1/2 (Figure 5C) [46] and collagenase inhibitor TIMP4.3 [47] genes, and these genes were overexpressed following injury (Supplementary Table S2).

Despite the obvious differences between mouse and zebrafish meninges, it is very informative to compare the patterns found in databases of RNA levels in mouse sections at Theiler stage 23 (E14.5) [48]) (Supplementary Figure S5B) with those in the Zfin database [49], an exceptional WMISH resource, particularly around 3 dpf (‘the high pec to long pec’ stage) (Supplementary Figure S5C). At this stage, the primordial cell types of the zebrafish meninges seem to be present, according to the observed patterns of expression for key genes (Supplementary Figure S5). Hence, despite the changes in zebrafish morphology between 3 dpf, 21 dpf, and the adult stage, the gene patterns observed at 3 dpf in the midbrain—our region of interest dissected out for scRNA seq—are similar to those of the ARA and MID populations of EGFP-positive cells in the midbrain at 21 dpf.

#### 3.2.5. Cell-Type Diversity within the OMN PDGFRβ Cluster

**Figure 7:**
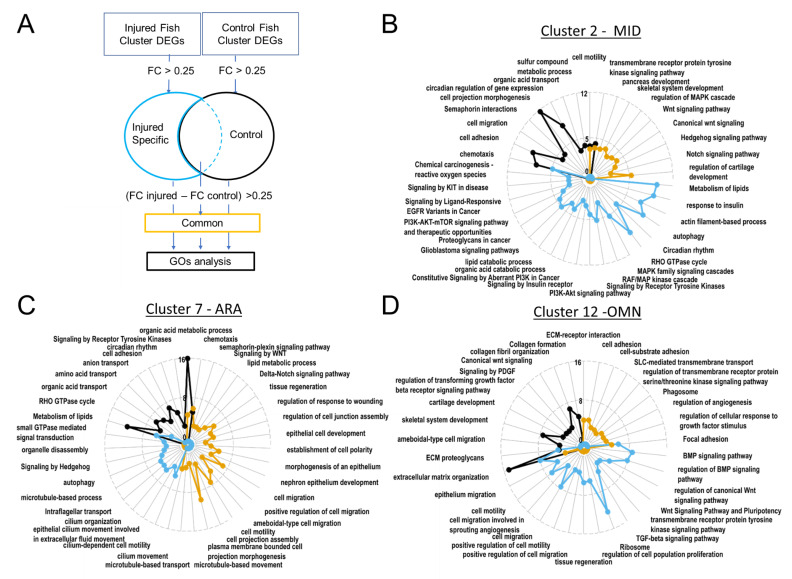
Gene ontology revealed upregulated biological processes. In ARA gene lists corresponding to genes specifically present in samples from injured fish, cilium-dependent cell motility GO and other cilium associated GOs are enriched. (**A**). Pipeline for filtering DEGs for GO analyses with Metascape. Radarplots of enriched Gene ontologies. Curves colored following types of filtering as explained in (**A**). Axis concern Zscore of GOs. Increased distance from the centre means higher significance. (**B**). OMN. (**C**). MID. (**D**). ARA.

We also focused on the small C16 cluster, which grouped together cells from control and injured juveniles. A very large proportion (89%) of the cells in this cluster corresponded to a subset of cells from injured fish. No corresponding cluster was visible in controls alone, but the small number of control fish cells represented made it impossible to compare the transcriptomes of control and injured subsets. Imaging revealed that meningeal fibroblasts were recruited to the wound. We therefore characterized this cluster by assuming that it contained this cell type, together with various meningeal cell types. We used a table listing the DEGs for each perivascular meningeal cell type in mice [42] to search for fish orthologs. We found that a surprisingly large number of genes was conserved. In particular, most OMN cells expressed PDGFRA, suggesting that they may have fibroblastic features, as already reported in the spinal cord [17,50,51]. The major component of C16 was a subset of cells producing large amounts of collagen (Figure 6). This may be a hallmark of pial cells or of other BBB perivascular, or possibly meningeal cells responsible for the secretion of the basal membrane in close contact with glial cells. However, in our imaging experiments, we did not detect any pial-like cells in juveniles, suggesting that these cell types are either poorly developed in juveniles or that they are not EGFP-positive. In control fish, an enrichment in GO terms relating to collagen secretion was observed in more than 50% of cells (Figure 7D). In injured fish cells, we also identified an enrichment in other ontologies relating to the extracellular matrix: cell substrate adhesion, focal adhesion, extracellular matrix organization, ECM receptor interaction, ECM proteoglycan, collagen formation, and collagen fibrin organization (Figure 7D). CLDN 11A/B were also found to be expressed in this cluster, with their levels of expression decreasing during injury (Figure 5C).

As reported in a previous study based on spinal cord injury [52], a strong enrichment in *col12a1a*COL12A1A expression was observed in injured OMN cells (Figure 6), whereas these cells had lower levels of LUMICAN (LUM) expression than control cells (Figure 5C). These findings suggest that ECM composition may change in OT fibroblasts, as already reported for spinal cord fibroblasts, to promote regeneration [52].

We also observed ligament and chondrocyte DEGs. We used COL2A1A and TMND, which are expressed in a mesenchymal ligament-like population above chondrocytes and characterized by MATN4 and GGCTB expression, to characterize head ligament cells. Enriched expression was observed for all these DEGs in about 5% to 20% of cluster 16 cells at 1 dpi (Figure 5C). This suggests that injury may have led to the recruitment of ligament and chondrocranium cells at this time point. ALPL, one of the few specific DEGs in mouse dura, was expressed in 10% of C16 cells.

Several DEGs were specific to C16. An enrichment in S100A10B filaments was observed in C16 OMN (Figure 6), whereas S100B/NESTIN enrichment was observed in MID and ARA (Figure 6). In addition, C16 cells expressed RBP4, a retinol transporter, and presented very high levels of expression for ADH8a (Figure 6), which may be responsible for the first step in retinoic acid synthesis, namely, the conversion of retinol into retinaldehyde. ARA and MID displayed a mild enrichment in ALDH1A2 (Figure 6), which is involved in the conversion of retinaldehyde into retinoic acid. Thus, as suggested in previous studies on mice [5,40], there may be a ‘division of labor’ within the meningeal layers of the zebrafish to ensure the provision of retinoic acid to the brain [5].

In this cluster, many signaling pathways seemed to be active after injury: the TGFb, WNT, and BMP signaling pathways (Figure 6, Supplementary Table S2). It was not, therefore, straightforward to determine, from this dataset, which pathways participated in cell recruitment and their potential roles in the recruitment of other cell layers for repair. Collagen-secreting cells increased proliferation after injury, as shown by the enrichment in expression for *ccn1* and other *ccn* genes, together with *jun* genes in about 50% of OMN cluster cells (Figure 5C).

In conclusion, the analysis of this cluster identified several cell types for the first analyses but also highlighted the difficulty achieving cell-type homology with such a small dataset. Much remains to be explored if we are to understand the diversity of perivascular, fibroblastic, and pial cells in zebrafish.

#### 3.2.6. The PI3K-AKT Pathway Is Active in MID Cells following Injury

The MID radar plot of enriched GO terms revealed the expression of many signaling pathways at homeostasis (canonical WNT, hedgehog, Notch) (Figure 7B). Based on GO term enrichment, these cells have metabolic features similar to those of ARA cells, such as the transformation of sulfur compounds or the transport of organic acids (Figure 7B,C).

In the subset of cells from injured juveniles at 1 dpi, we detected enrichment in activated tyrosine kinase receptors and the PI3K-AKT and MAPK pathways, which transduce tyrosine kinase receptor signals. Enrichment was also observed for many GOs involved in cancers (and, more specifically, metastatic processes; Figure 7B). However, these cells did not express common markers of proliferation, such as bub1, MCM2/6, CCN, CCNB/D/E, E2F1, FOXM1, JUN, MYBL2, PLK1, or TOP2 [53], suggesting that these cells become motile without proliferating after injury.

In conclusion, these cells probably activate the AKT pathway post-injury in response to a tyrosine kinase, which could, for example, be PDGFRβ, which is also involved in activating proliferation and motility in the endothelium [45]. This suggests that cells are probably recruited as ARA. Our imaging experiments unambiguously identified waves of migration from the midline (Figure 4A,D,G,J).

#### 3.2.7. ARA Boosts the Ciliary Machinery

Several GO terms relating to cell migration (including terms relating to projection migration) displayed enrichment in the ARA subset corresponding to injured fish (Figure 7C). The Wnt/PCP and Hedgehog signaling-associated GO terms also displayed strong enrichment in injured fish ARA cells (Figure 7C). These signaling systems play a crucial role in regulating the directionality, speed, and persistence of cell migration. Hedgehog involves the cilium transduction hub and intra-flagellar transport, for which the associated GO terms displayed enrichment in cells from injured fish (Figure 7C).

**Figure 8:**
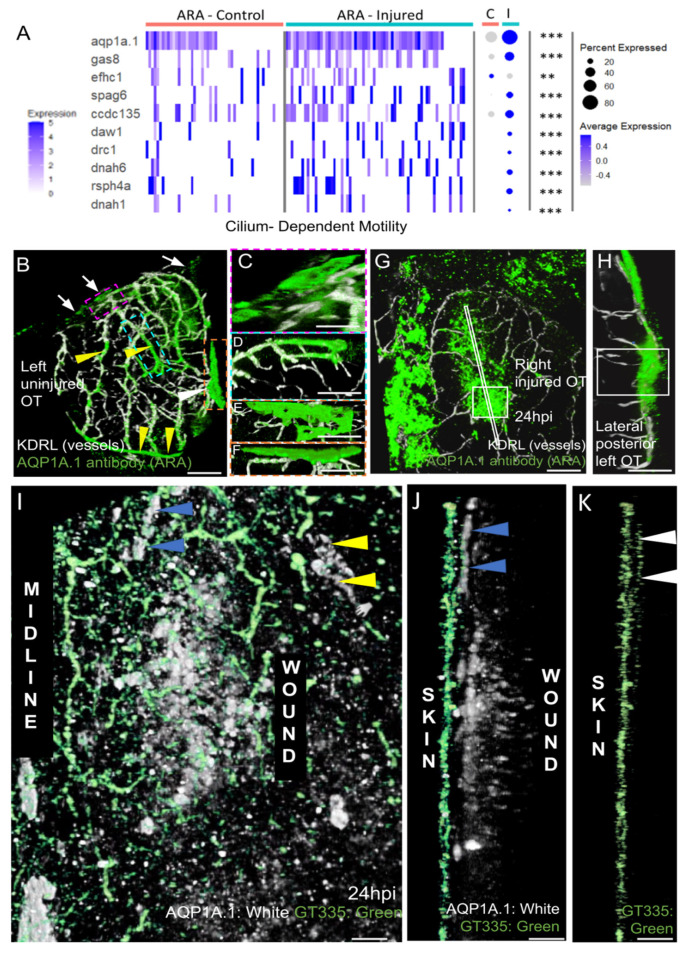
AQP1a.1 ciliated ARA migrates towards the wound. (**A**) Expression of AQP1a.1 and cilia motility genes in cells depending on their status show overexpression of these genes after wounds. Genes expressed <10% of cells are not presented in dotplot. Adjusted *p* value (** <0.01 *** <0.001) concern markers in injured status. (**B**) Dorsal view on an uninjured OT with ARA (white arrows), MID (white arrowhead), and vessels (yellow arrowheads) stained by the AQP1a.1 antibody. Anterior at the top. Midline on the left. (**C**) Detailed dorsal view of ARA. (**D**) Detailed side view of ARA. (**E**) Detailed dorsal view of MID. (**F**) Detailed side view of MID. (**G**) At one dpi, strongly positive AQP1a.1 cells are visible over the wound (white arrowheads). (**H**) Strong and thick AQP1a.1 staining over the wound. (**I**) AQP1a.1-positive cells over and around the wound. Peripheral ARA-like cells: yellow arrowheads. (**J**) Migrating ARA stained by AQP1a.1: blue arrowheads. Lateral ARA: yellow arrowheads. (**K**) Migrating AQP1a.1-positive ARA are ciliated as shown by the GT335 staining: white arrowheads. AQP1a.1-positive cells do not exhibit any cilium/GT335 staining. Scale bars: (**A**,**F**): 100 μm; (**B**–**E**,**G**): 50 μm; (**H**–**J**): 10 μm.

Most strikingly, our analysis of GO term enrichment in ARA cells from injured fish clearly highlighted a strong enrichment of cilium-dependent motility genes (Figure 8A). Indeed, 9 of 35 genes associated with ciliary motility were significantly more strongly expressed in cells originating from injured fish (Figure 8A).

Strong enrichment was also observed for *FoxJ1A* during regeneration (Figure 5C). At 6 hpi, enrichment was maximum (log2 FC: 3.7) (not shown) at a time when there were numerous migrating cells (not shown). This gene is known to be a master gene, regulating ciliary function in the epidermis during regeneration [54].

More specifically, we observed the increase of motile cilia markers. Among them, dynein arms (DNAH1, DNAAH6, DNAAF1, DNAAF11, ODAD1, ODAD3) and radial spokes (RSPH4A) found on the central pair of microtubules characterize most motile cilia. Expressions of general cilia markers, such as IFTs, were also increased. Motile cilia can be present in a single copy on the cell surface, as in renal epithelia, or in the node defining laterality, or in multiple copies as in multi-ciliated cells.

To produce a large quantity of new multi-ciliated cells (with 30 or 100 cilia), it is necessary to multiply the centrioles that serve as anchoring points for the cilia in post-mitotic cells by boosting the activity of very specific proteins: Multicilin or MCC, which transcriptionally activates genes for basal body production and the gene for FoxJ1a, the master regulator for basal body docking [54], cilia formation, and motility; geminin (GEMC), which regulates MCC [55]; DEUP1; PLK4; SAS6; and CEP152 and CDC20B, involved in centriole biogenesis. We did not find an increase in expression for any of these genes. This probably means that ARA cells are single-ciliated with a motile cilium, and with a central pair (a structure sometimes called 9 + 2 [56]. This type of cilium combines a function of fluid movement but also of signal transduction.

#### 3.2.8. After Injury, AQP1a.1-Positive Cells Activate the Cilium Machinery and Accumulate over the Wound

In mammals, AQP1 plays a key role in CSF production and homeostasis [29]. In zebrafish larvae, AQP1a.1 facilitates the movement of CO_2_ and ammonia [22]. Mouse scRNAseq data suggest that this gene is not differentially expressed in the various layers of the meninges [40], and WMISH data have revealed a broad range of expression in meningeal cells [48]. Our data revealed that this was one of the few MID-ARA DEGs for which expression increased during regeneration (Figure 5C, Figure 6 and Figure 8A). We therefore investigated the distribution of the AQP1A.1 protein during regeneration.

In control fish, we first used an antibody that gave peripheral staining in ARA, particularly over the putative subarachnoid cisterns, which are adjacent to the cerebellum and telencephalon in mice [57]. Staining was also detected at the midline in MID (Figure 8B,E,F) and in the OT vessels (Figure 8B). Positive signals were detected in peripheral leptomeningeal vessels, as in clarified mouse brain [57]. Intriguingly, this antibody pattern differed from the restricted meningeal pattern of expression observed at an earlier stage by WMISH [48].

As expected, a strong AQP1 antibody signal was observed at the wound at 1 dpi (Figure 8G,H). The pattern of AQP1a.1 staining (Figure 8G), thus, coincided with the distribution of EGFP-positive cells observed at the wound (Figure 4).

Finally, based on our scRNAseq results, which indicated amplification of the ciliary machinery, promoting motility, we used a mouse GT335 antibody to detect cilia. Strong signals were detected in the skin (Figure 8J), as reported in mammals [58]. By contrast, no cilia were detected on the AQP1a.1-positive cells located at the wound (Figure 8J), whereas a high density of cilia was observed in the ARA-like cells at the periphery of the wound (Figure 8K). These data are consistent with our scRNAseq results, suggesting a significant increase in the transcription of ciliary genes in ARA cluster cells after injury.

## 4. Conclusions

This study focused on a scRNA-seq analysis coupled to high-resolution 3D imaging on zebrafish midbrain meningeal cell types in homeostasis or after OT injury. This approach made it possible to determine which cell types were involved in repair and to identify the mechanisms potentially activated. Overall, we observed a surprising conservation of pathways and genes expressed in both zebrafish and mouse meningeal clusters. In particular, we were able to identify arachnoid markers conserved between zebrafish and mice and possible candidate genes for cell recruitment for meninx repair. This study therefore provides insight into meningeal recovery after nervous system surgery or trauma, together with meningeal cysts and cancers. For example, mechanisms activated during MID migration may be dysregulated in meningiomas. This study therefore opens up new avenues in the study of various pathological aspects of the meninges. Using our ZebrafishBrainInspector (ZBI) [59] fast imaging platform, we will be able to develop a novel screening pipeline for candidate genes or molecules affecting the behavior of meningeal cells.

## Data Availability

Not applicable.

## Supplementary Materials

TThe following supporting information can be downloaded at: https://www.mdpi.com/article/10.3390/cells11132016/s1, Figure S1: Supplemental scRNAseq analysis; Figure S2: Needle injury of the optic tectum (OT) at 21 dpf; Figure S3: Deep laser injury of the OT; Figure S4: Gating strategy for EGFP-positive cell sorting; Figure S5: Selected WMISH patterns of expression (Zfin) at 3 dpi of hallmark genes of the twelve clusters in the UMAP; Figure S6: Examples of meningeal patterns obtained from control fish, Table S1: Control fish DEGs, Table S2: Merged control/injured fish DEGs.
