# Supplementary material for: Molecular and Cellular Analysis of the Repair of Zebrafish Optic Tectum Meninges Following Laser Injury
Source: Cells. 2022 Jun 24;11(13):2016. doi: 10.3390/cells11132016 (PMC9266167; doi:10.3390/cells11132016)
Supplement: Supplementary file 1 [file cells-11-02016-s001.zip › cells-1724495-Supplementary-figures.pdf]

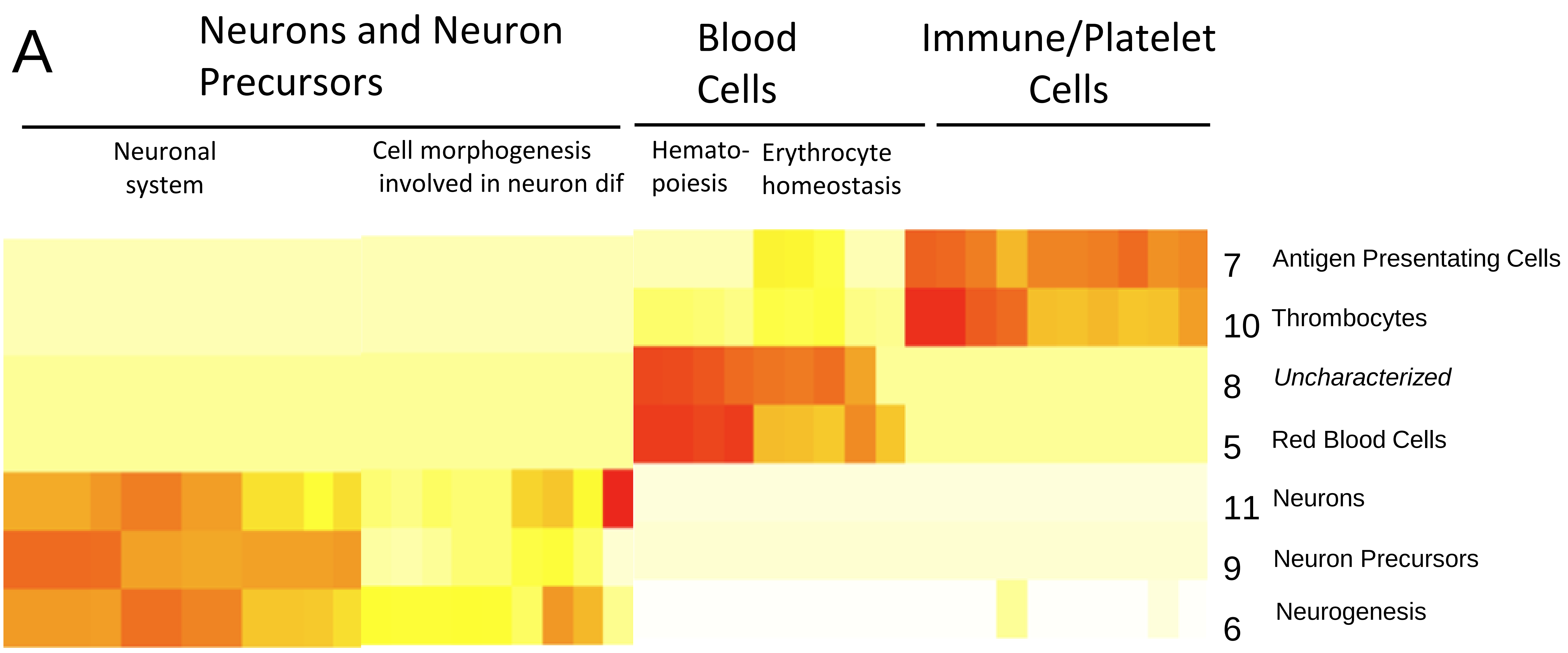

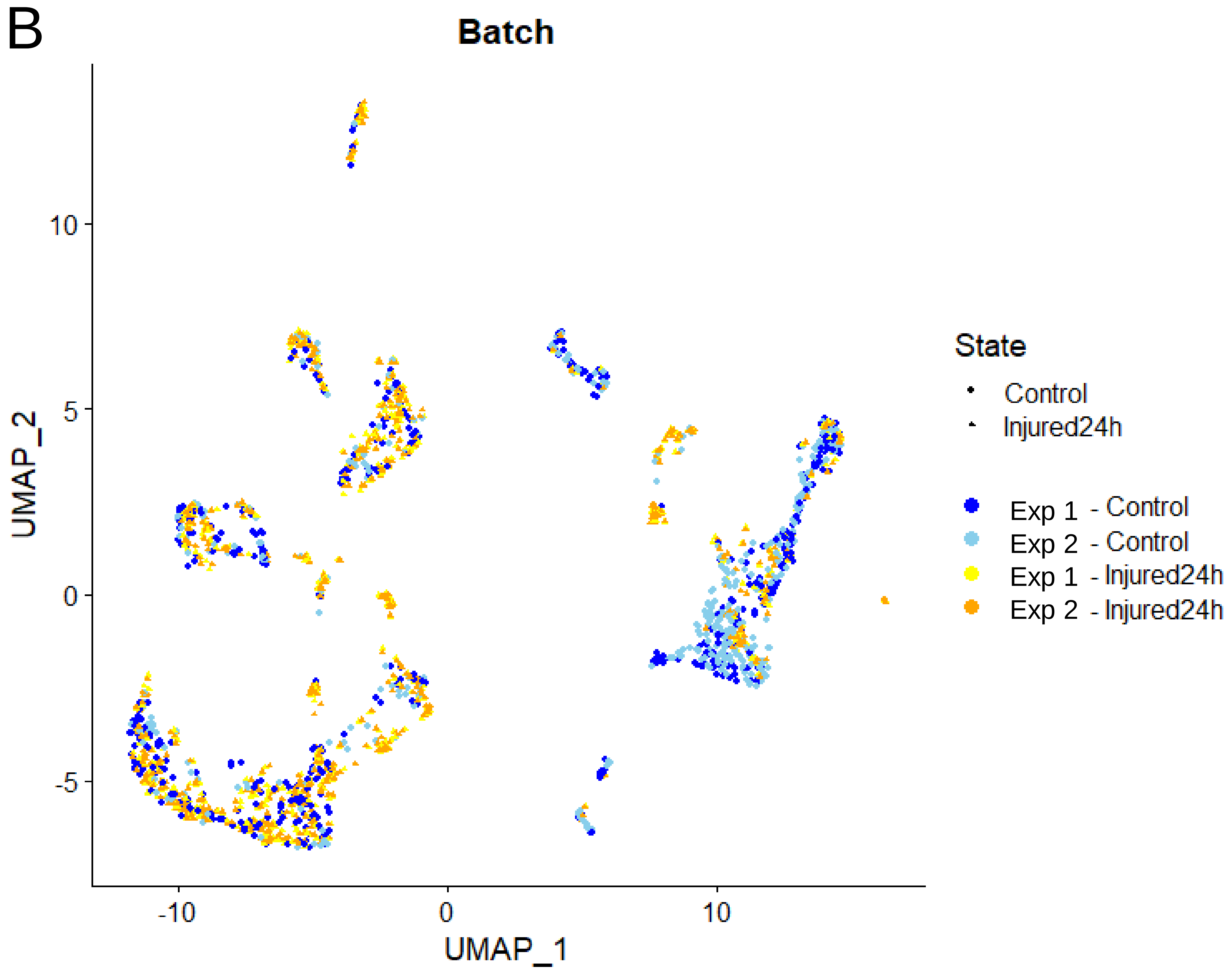

Supplementary Figure S1. Supplemental scRNAseq analysis. (A) Heatmap of clusters from control fish identified on the basis of GO enrichments. On top are summarised types of GOs. Cluster identifications are indicated on the right. Clusters are grouped according to convergent GOs. Red: strong enrichment. Orange: mild enrichment. Yellow/white: weak or no enrichment. **(B)** UMAP projection of cells colored depending on their sample origin.

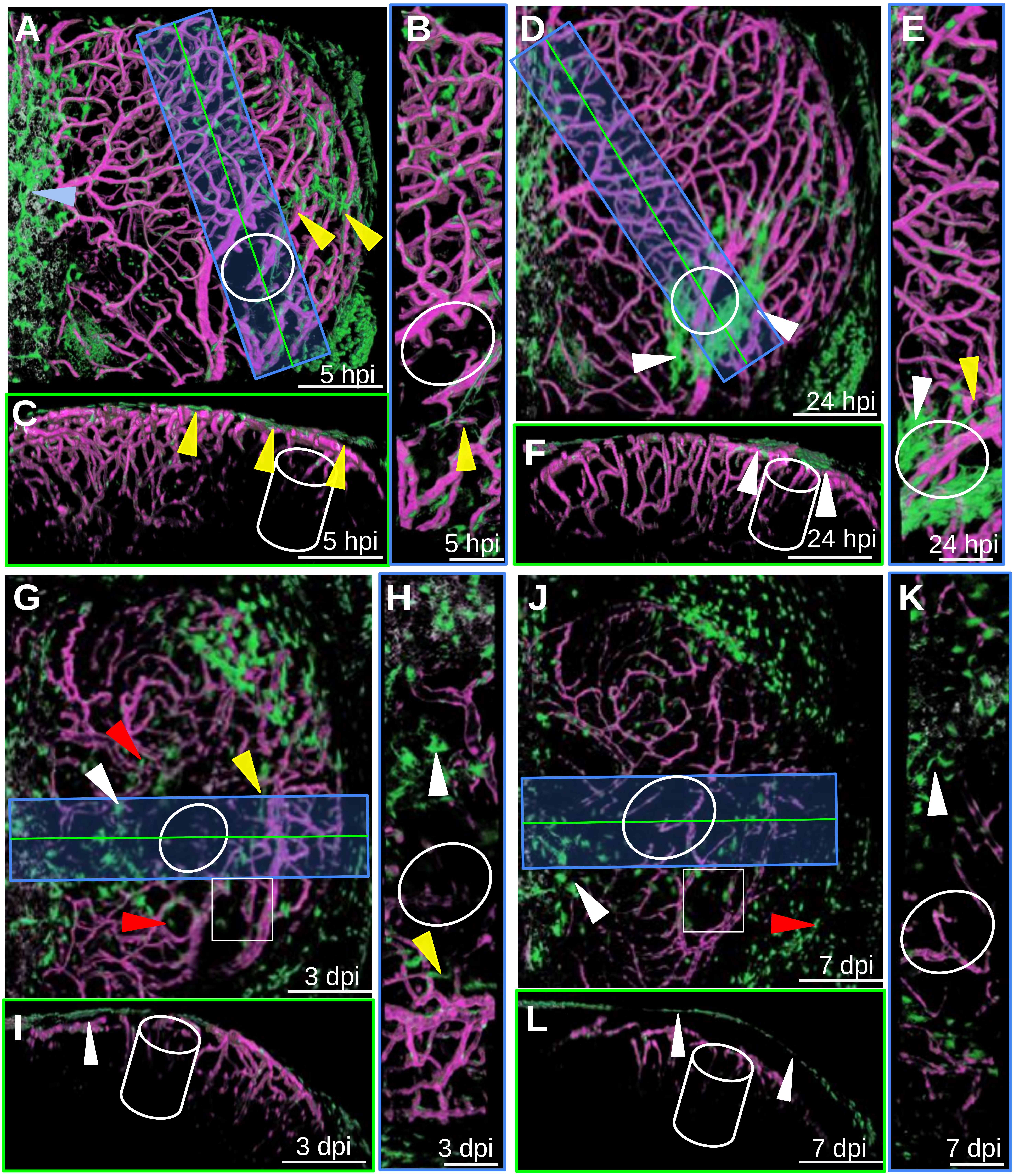

Supplementary Figure S2. Needle injury of the optic tectum (OT) at 21dpf. Cells are fastly recruited to the wound. (**A, D, G, J**). Dorsal views. White circles and cylinders: locations of injury visible up to one dpi. Anterior at the top. Midline on the left. (**B, E, H, K**) Horizontal thick sections. (**C, F, I, L**) XZ sections. (**F**). At one dpi, multiple layers of arachnoid cells are visible over the wound (white arrowhead). (**I**). EGFP positive cells spread over the OT (white arrowheads). (**L**). EGFP form a quasi-uniform layer over the OT. Putative injured zones: white square. ARA-like cells: yellow arrowheads. MID-like cells: blue arrowheads. Fibroblast-like cells: red arrows.Scale bars: (A, C, G, I, D, F, J, L): 100 µm. (B,E,H, K): 50µm.

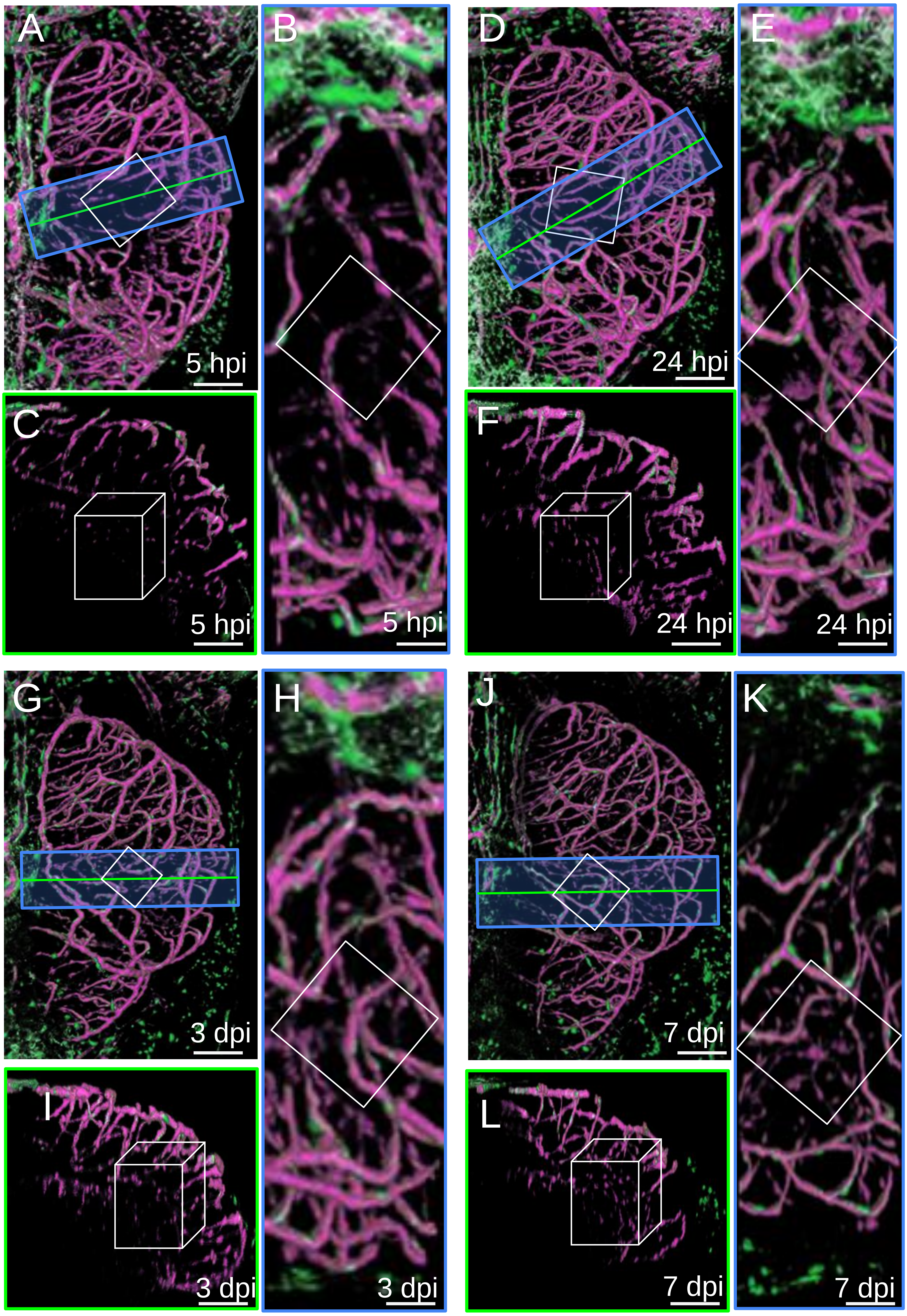

Supplementary Figure S3. Deep laser injury of the OT. This type of injury led to no massive cell recruitment of pericytes or other cell types at 21dpf. (**A, D, G, J**). Dorsal views. Anterior at the top. Midline on the left. (**B, E, H, K**) Horizontal thick sections. (**C, F, I, L**) XZ sections. Whites squares and parallelepipeds: locations of injury. Scale bars: (A,C,D,F,G,I,J,L): 100 µm. (B,E,H,K): 50 µm.

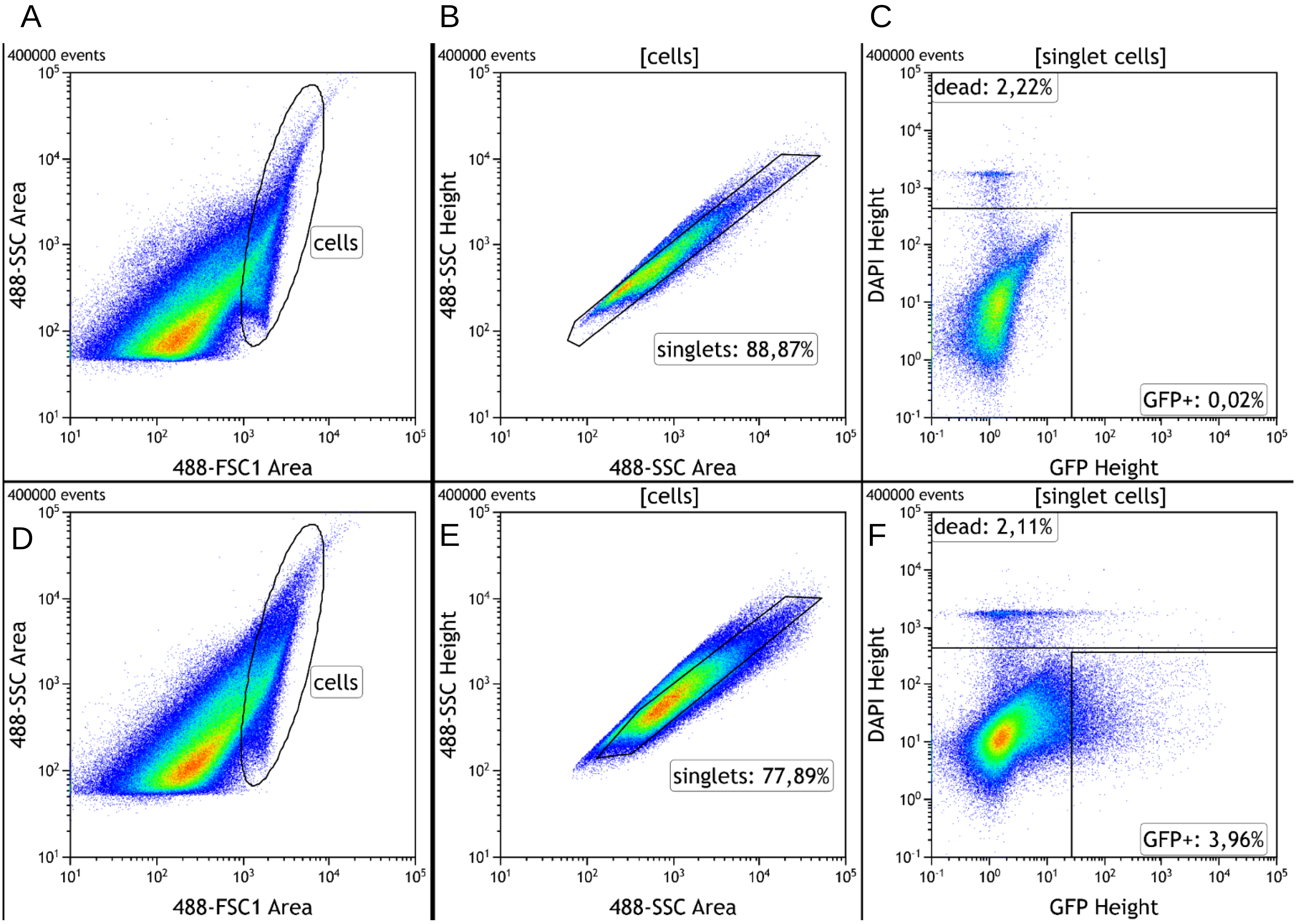

Supplementary Figure S4: **Gating strategy for EGFP-positive cell sorting. (A, B, D, E)**. Selection of cell populations. **(C, E)** Selection of singlets with DAPI staining. **(A, B).** Cell sorting from non transgenic wild-type fish. **(C)**. No EGFP positive cell was sorted in control wild-types.**(D-F)** Cell sorting from injured dissected midbrains of the PDGFRB-gal4:UAS-GFP at one day post-injury. **(F)**. 4% of singlets were EGFP positive.

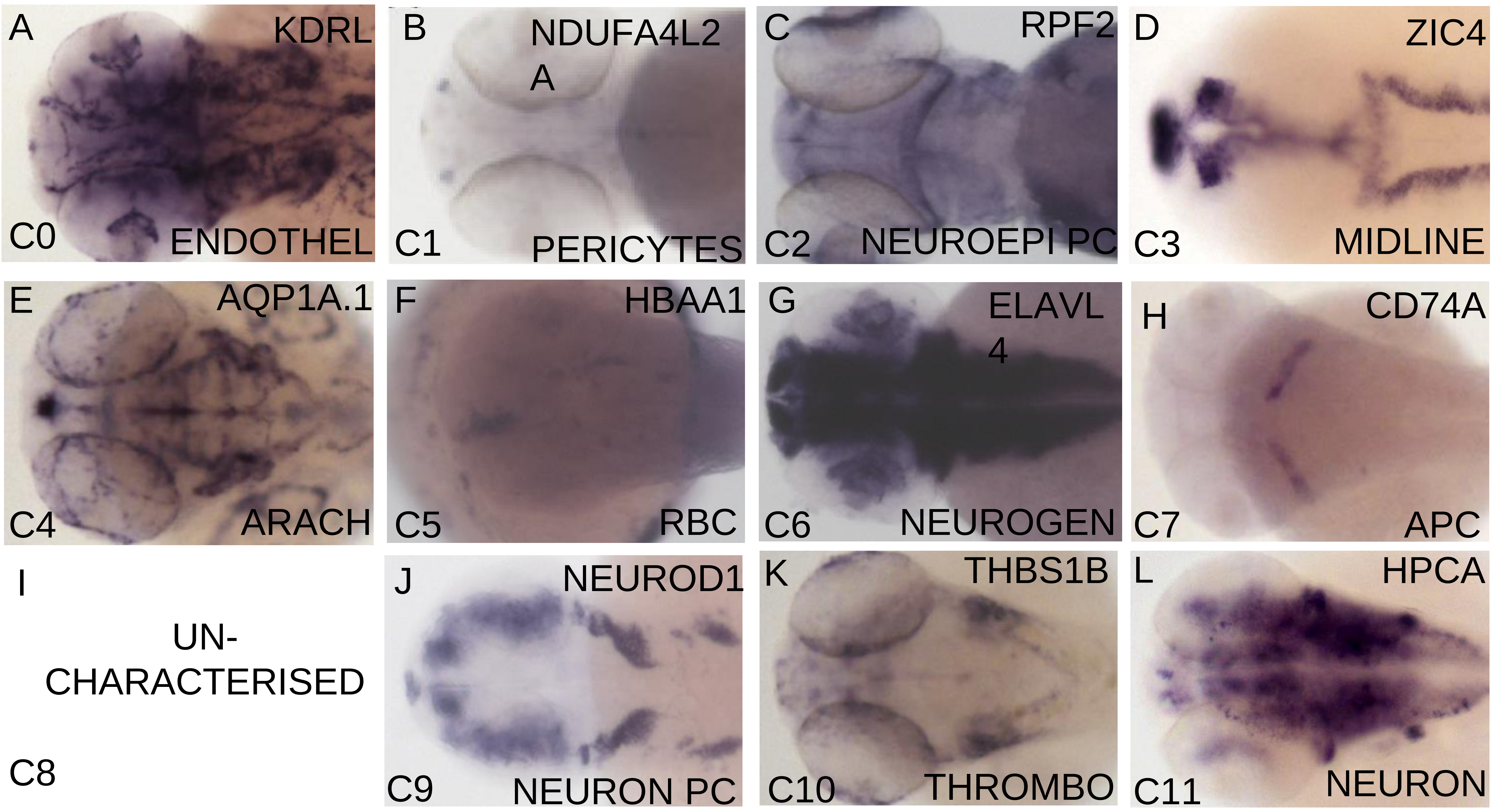

Supplementary Figure S5. Selected WMISH patterns of expression (Zfin) at 3dpi of hallmark genes of the twelve clusters in the UMAP computed from control fish dataset (Figure 2B). Names of the genes are indicated at the top right of each picture. They are chosen among most strongly enriched DEGs. Names of the clusters are indicated below on the right. Numbers of the clusters are below left.

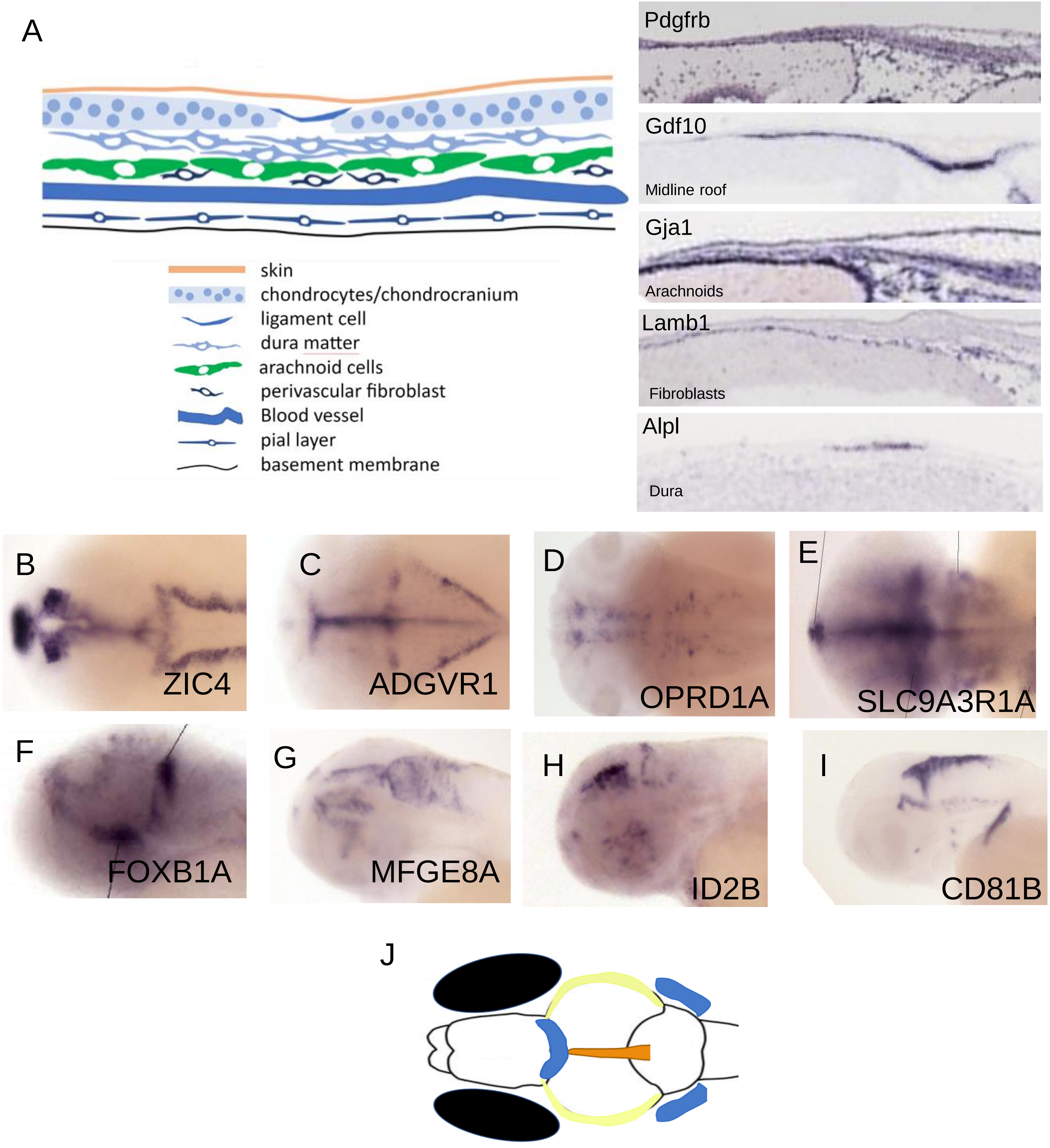

Supplementary Figure S6. Examples of meningeal patterns. (**A**). Left panel: Schematic diagram of main meningeal layers in mice. Right panel: patterns of expression of DEGs of main meningeal layers extracted from Eurexpress/JGI data (MGI database) (**B**). Patterns of expression by WMISH at 3dpi extracted from Zfin of enriched genes in the midiline roof (MID) cells (B,E) and arachnoid (ARA) cells (F,I). At this early stage, MID patterns already stain dorsal midline. ARA exhibit latero-dorsal expression patterns as in 21 dpf juveniles. (**J**). Schematic diagram of MID (yellow), ARA (orange) and RBC (blue) domains at 21dpf similar to WMISH patterns at 3dpf.
